# Association between *AXIN1* Gene Polymorphisms and Bladder Cancer in Chinese Han Population

**DOI:** 10.1155/2019/3949343

**Published:** 2019-04-15

**Authors:** Qin Li, Peng Zhang, Yanyun Wang, Yan Zhang, Kai Li, Yaping Song, Min Su, Bin Zhou, Lin Zhang

**Affiliations:** ^1^Laboratory of Molecular Translational Medicine, Center for Translational Medicine, Key Laboratory of Birth Defects and Related Diseases of Women and Children (Sichuan University), Ministry of Education, West China Second University Hospital, Sichuan University, Chengdu, Sichuan 610041, China; ^2^Department of Immunology, West China School of Preclinical and Forensic Medicine, Sichuan University, Chengdu, Sichuan 610041, China; ^3^Department of Urology, West China Hospital, Sichuan University, Chengdu, Sichuan, China; ^4^Department of Pathology, West China Second University Hospital, Sichuan University, Chengdu, Sichuan 610041, China; ^5^Department of Cardiology, West China Hospital, Sichuan University, Chengdu, Sichuan 610041, China

## Abstract

**Background:**

Previous evidence has indicated that the reduction of axis inhibition protein 1 (*AXIN1*) expression is related with the poor differentiation of non-small-cell lung cancer (NSCLC). However, the potential association between AXIN1 and bladder cancer (BC) is unknown. We aimed to initially explore the relevance of *AXIN1* gene polymorphisms (rs12921862 C/A, rs1805105 T/C, and rs370681 C/T) and BC.

**Methods:**

Three hundred and sixteen BC patients and 419 healthy controls had been enrolled. Polymerase chain reaction-restriction fragment length polymorphism (PCR-RFLP) method was used for genotyping three tag single-nucleotide polymorphisms (SNPs) of *AXIN1*. The SNPstats online analysis software and SPSS software were used for statistical analysis.

**Results:**

Our data revealed that three tag SNPs were associated with an increased risk of BC (rs12921862: *P* < 0.001, OR (95%CI) = 4.61 (3.13-6.81); rs1805105: *P* = 0.046, OR (95%CI) = 1.35 (1.00-1.82); and rs370681: *P* = 0.004, OR (95%CI) = 1.56 (1.15-2.10)). For rs12921862, A allele was an independently protective factor which correlated with a better prognosis in non-muscle-invasive bladder cancer (NMIBC) patients (*P* = 0.03, OR (95%CI) = 0.10 (0.01-0.84)). Stratification analysis demonstrated that rs370681 polymorphism was related with high-grade bladder cancer (*P* = 0.04, OR (95%CI) = 1.85 (1.04-3.23)).

**Conclusion:**

The *AXIN1* gene polymorphisms might implicate in BC risk, and rs12921862 could be a potential forecasting factor for prognosis of BC patients.

## 1. Introduction

Bladder cancer (BC) was ranked as the ninth most common cancer by the International Agency for Research on Cancer in 2012. It is related to thrice as much higher morbidity and mortality in the developed countries compared to the developing regions, and more than 75% of the cases occur in men. In 2012, 165000 deaths and 429000 new cases were recorded worldwide, of which 26820 deaths and 55486 new cases were in China, resulting in an incidence rate of 3/100000 and accounting for a large fraction of BC in East Asia (37491 deaths and 85451 cases) [1]. Around 80% of BC patients have the non-muscle-invasive bladder cancer (NMIBC), whereas muscle-invasive bladder cancers (MIBC) account for only 20% of the cases, although they are responsible for a large number of deaths [2–4].

Smoking is the most significant risk factor of BC, accounting for an estimated 50% of all cases, followed by occupational exposure to chemical carcinogens, e.g., industrial paints, dyes, metals, and petroleum products [3–5]. Increasing evidences in recent years have suggested a genetic predisposition towards cancer susceptibility. For instance, Gu et al. found an association between the N-acetyl transferase 2 (NAT2) slow acetylator phenotype and a significantly higher risk of BC in smokers [6]. Moreover, the risk of BC is twofold higher in first-degree relatives of BC patients [4], indicating that several genetic factors could play a role in the initiation and progression of BC.

The Wnt/*β*-catenin signaling pathway is one of the fundamental pathways regulating cell proliferation, polarity, and lineage differentiation during embryonic development and tissue homeostasis, and mutations in its components are often involved in birth defects, cancer, and other diseases [7, 8]. Studies have directly linked the Wnt signaling pathway to the development of BC [9–11]. Mao et al. demonstrated that activating the Wnt pathway could accelerate the epithelial-mesenchymal transition (EMT), invasion, and migration of BC cells *in vitro* [12]. In addition, the inhibition of Wnt signaling suppressed BC xenograft growth in nude mice [13]. Canonical Wnt signaling is regulated by the degradation of the AXIN1-mediated *β*-catenin destruction complex. Axis inhibition protein 1 (AXIN1) is a multidomain scaffold protein that regulates the levels and localization of *β*-catenin during Wnt pathway activation and is involved in the genesis and progression of diseases like atrial septal defect, cryptorchidism, caudal duplication anomalies, breast cancer, non-small-cell lung cancer (NSCLC), hepatitis B virus-related hepatocellular carcinoma (HCC), colorectal cancer, and gastrointestinal cancer [14–22].

*AXIN1*, the gene encoding AXIN1, is located within a 65 kb region on chromosome 16p. AXIN1 acts as a tumor suppressor, and mutations in this protein have been shown to play a significant role in carcinogenesis [23]. However, no link has been established so far between AXIN1 and BC. The aim of our study was to determine whether the polymorphic variants of *AXIN1* contribute to BC susceptibility. We selected three tag single-nucleotide polymorphisms (SNPs)—rs12921862, rs1805105, and rs370681—and determined their prevalence in 316 unrelated BC patients and 419 healthy controls in a Chinese Han population.

## 2. Material and Methods

### 2.1. Participants' Clinical Characteristics and Follow-Up

We enrolled 316 patients with bladder cancer (mean age ± SD: 63.76 ± 12.14) and 419 healthy controls (mean age ± SD: 59.88 ± 11.32) from the West China Hospital of Sichuan University from 2007 to 2012. A case control study based on the hospital was approved by the hospital ethics committee, and informed consents were provided by all the participants. Follow-up data of participants were abstracted by telephone calls every 6 months for 5 years. Histopathological analysis was used to confirm the tumor tissues from resected specimens of patients, and the clinical characteristics are summarized in Table 1. This study excluded the control participants who had a personal or family history of BC or other severe diseases and the patients who had previous cancer or metastasized cancer from other origins as well as underwent radiotherapy or chemotherapy. All participants were genetically unrelated individuals of the Han population living in Sichuan province of China.

### 2.2. AXIN1 Genotyping

The three tag SNPs were picked out according to data in the CHB population sample of the HapMap Project (Data Release 24/Phase II, NCBI build 36 assembly, dpSNPb126) using the algorithm-Tagger-pairwise Tagging from the international HapMap Project [24]. And the PCR primers were designed with software Primer 3 web version 4.1.0. (http://primer3.ut.ee/) [25] as shown in Table 2 (in this part, we followed the methods of Li et al. [26]).

DNA isolation kit from BioTeke (Peking, China) was used to extract each individual's genomic DNA from a 200 *μ*L EDTA-anticoagulated peripheral blood sample. Genotyping was performed using polymerase chain reaction-restriction fragment length polymorphism (PCR-RFLP). The DNA fragments containing the polymorphisms were amplified in a total volume of 10 *μ*L, including 5 *μ*L of 2x power Taq PCR Master Mix (BioTeke, Peking, China), 2.7 pico mole of each primer, and 100 ng genomic DNA for rs12921862 and rs1805105, and a total volume of 25 *μ*L, including 2.5 *μ*L of Taq Buffer, 0.3 *μ*L Taq enzyme (BioTeke, Peking, China), 3 *μ*L DNTP, 2.7 pico mole of each primer, and 100 ng genomic DNA for rs370681. The PCR conditions were 94°C for 4 min, followed by 34 cycles of 30 s at 94°C, 30 s at 62°C, 65°C, or 66°C, respectively, and 30 s at 72°C, with a final elongation at 72°C for 10 min for three tag SNPs. Following the termination of PCR, the PCR products were digested by restriction enzyme (presented in Table 2). Finally, the digested fragments were separated by a 6% polyacrylamide gel and stained with 1.5 g/L argent nitrate. Furthermore, the genotypes were confirmed by DNA sequencing analysis. About 10% of the samples were randomly selected to perform the repeated assays, and the results were 100% in agreement.

### 2.3. Statistical Analyses

SPSS for Windows software package version 20.0 (SPSS Inc., Chicago, IL, USA) was used to analysis the data. Genotypic association were provided by the SNPstats online analysis software including the codominant, dominant, recessive, and overdominant genetic models [27], and the Hardy-Weinberg equilibrium was evaluated by chi-squared test. The level of significance was set at *P* < 0.05. The effects of different genotypes and alleles were evaluated by odds ratio (OR) and respective 95% confidence intervals (95% CI). Kaplan-Meier plots and the log-rank test were used to estimate the relationships of *AXIN1* genotypes with patients' outcomes (recurrence and death). Considering age at first diagnosis, sex, smoking status, tumor grade, and clinical stage, Cox regression analysis model was used for multivariate survival analysis.

## 3. Results

### 3.1. AXIN1 SNP Frequencies and BC Susceptibility

The distributions of the three tag SNPs' genotypes and alleles in both case and control groups were in agreement with the Hardy-Weinberg equilibrium (*P* > 0.05). The differences in the genotypes and allele frequencies of *AXIN1* SNPs between patients and controls are shown in Table 3. The frequency of the heterozygous genotype (CA) of rs12921862 was significantly higher among the patients compared to controls in the overdominant model (33.5% vs. 8.8%, *P* < 0.001, OR = 5.21, 95% CI = 3.46 − 7.86). In the dominant model, a significantly increased BC risk was related to the CA/AA genotypes (35.1% vs. 10.5%, *P* < 0.001, OR = 4.61, 95% CI = 3.13 − 6.81). Compared to the CC/AA genotypes, the CA genotype was associated with a higher risk of BC in the codominant model (*P* < 0.001, OR = 5.24, 95% CI = 3.47 − 7.91). Similarly, a significant relationship was observed between the A allele-carrying patients and controls (18.4% vs. 6.1%, *P* < 0.001, OR = 3.45, 95% CI = 2.44 − 5.00). For rs1805105, compared with TT carriers, TC/CC carriers were significantly higher in the dominant model (46.5% vs. 39.1%, *P* = 0.046, OR = 1.35, 95% CI = 1.00 − 1.82). In the overdominant model, the TC genotype was significantly associated with increased BC risk compared to the TT/CC genotypes (*P* = 0.04, OR = 1.38, 95% CI = 1.01 − 1.87). For rs370681, significant differences were found in the codominant (CC vs. CT vs. TT, *P* = 0.002, OR_CT_ = 1.43, 95% CI_CT_ = 1.05 − 1.96, OR_TT_ = 2.51, 95% CI_TT_ = 1.43 − 4.40), dominant (CC vs. CT/TT, *P* = 0.004, OR = 1.56, 95% CI = 1.15 − 2.10), and recessive models (CC/CT vs. TT, *P* = 0.01, OR = 2.07, 95% CI = 1.21 − 3.55) compared to healthy controls. Furthermore, the frequency of T allele was significantly higher in the BC patients compared to the healthy controls (*P* = 0.001, OR = 1.46, 95% CI = 1.16 − 1.82).

### 3.2. Clinical Characteristics of the SNP Genotypes

To gain further insights into the association between the three tag SNPs and BC, the patients with different SNP genotypes were stratified (Table 4) according to age (≤64 and >64 years old), sex (male and female), smoking status (smoker and nonsmoker), tumor stage (MIBC and NMIBC), tumor grade (high grade and low grade), recurrence status (recurrent and nonrecurrent), and metastasis status (metastatic and nonmetastatic). No significant difference was observed for any subgroups of the three tag SNPs except age and tumor grades. The CA/AA genotypes of rs12921862 had a significantly higher percentage of patients older than 64 years compared with those under 64 years (40.1% vs. 29.2%, *P* = 0.04, OR = 1.64, 95% CI = 1.01 − 2.70), after adjusting for sex, smoking status, tumor stage, tumor grade, recurrence status, and metastasis status. For rs370681, after adjusting for these common risk factors, the T allele-carrying subgroup had a higher frequency of patients with high-grade tumors compared to those with low-grade tumors (68% vs. 54%, *P* = 0.04, OR = 1.85, 95% CI = 1.04 − 3.23). In contrast, no significant relation was seen between the rs1805105 genotypes and the patients' characteristics after adjusting for common risk factors (*P* > 0.05).

### 3.3. Prognostic Value of the SNP Genotypes

**Figure 1:**
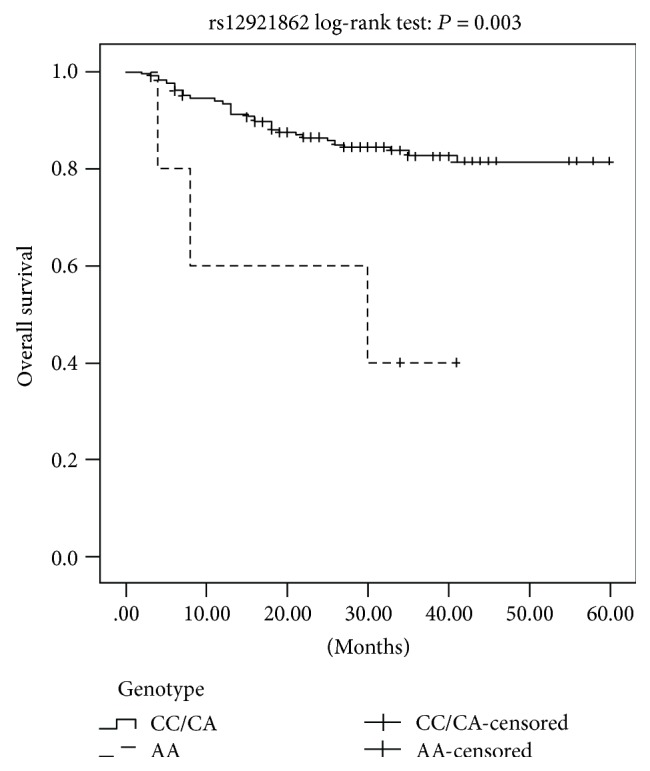
Kaplan-Meier survival curves for the recessive model of *AXIN1* rs12921862 polymorphism in BC patients.

The statistical association of the SNPs and overall survival of BC patients is summarized in Table 5. At the end of the follow-up in our study, 51 patients (51/316; 16.1%) including 38 MIBC patients and 13 NMIBC patients had died and 93 patients (93/316; 29.4%) including 46 MIBC patients and 47 NMIBC patients had relapsed. Kaplan-Meier survival analysis indicated worse prognosis of BC patients with the rs12921862 AA homozygote (log-rank: *P* = 0.003, Figure 1). However, no significant relationship was observed between the overall survival and the other two SNPs (rs1805105 and rs370681) or between the three SNPs and recurrence-free survival.

**Figure 2:**
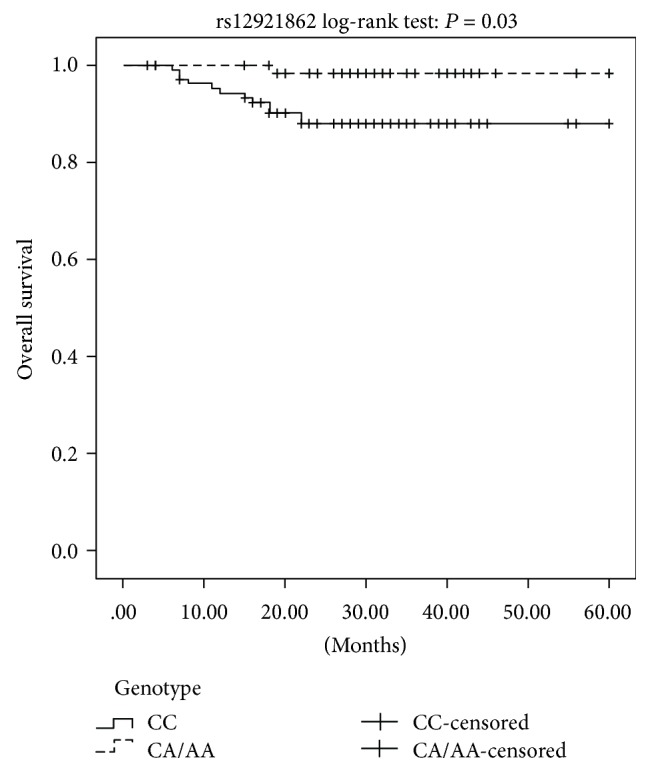
Kaplan-Meier survival curves for the dominant model of *AXIN1* rs12921862 polymorphism in NMIBC patients.

Following the stratification of patients by tumor stage (MIBC and NMIBC), we conducted Cox univariate and multivariate analyses to determine the predictors of prognosis and survival in the different SNP genotypes (Table 5). Univariate analysis showed no significant association between tumor stage and overall survival in any genotype of the three SNPs in BC patients. However, the multivariate analysis showed a correlation between the CA/AA genotypes of *AXIN1* rs12921862 and significantly higher overall survival rate (*P* = 0.03, OR = 0.1, 95% CI = 0.01 − 0.84), compared to the CC genotype in NMIBC patients. Kaplan-Meier survival analysis reiterated that the CA/AA genotypes were associated with a better prognosis of NMIBC patients (log-rank: *P* = 0.03, Figure 2) compared to the CC genotype.

## 4. Discussion

The Wnt signaling pathway plays a key role during embryonic development and tissue homeostasis, and its dysregulation is associated with various diseases including cancers [7, 8]. The tumor suppressor function of AXIN1 and AXIN2 proteins in the Wnt signaling pathway has long been hypothesized, based largely on their roles in the *β*-catenin destruction complex. However, no study had directly linked AXIN1 with BC progression or the proliferation of BC cells. In the present study, we analyzed the potential relationship between different *AXIN1* gene SNPs and BC risk in a Chinese Han population. Despite extensive linkage disequilibrium in the human genome, a tag SNP could represent most of the haplotypes in a specific region. Our data revealed that the three tag SNPs were associated with an increased risk of BC, which corresponded to the previous observations that activating or inhibiting Wnt signaling accelerates or suppresses, respectively, the invasion and migration of BC cells as well as the carcinogenesis of BC tumor [12, 13]. We calculated the statistical power using Power and Sample Size Calculations for Windows software package version 3.0 [28], and it showed that our data had more than 80% power to detect the relationship between *AXIN1* SNPs and BC risk.

Tumor suppressor protein AXIN1 deregulates *β*-catenin and mitosis to weaken the ER^+^ and ER^−^ breast cancer [14, 19]. It interacts with *β*-catenin and regulates its localization, along with that of Wnt-dependent downstream targets like cyclin D1, fra-1, c-myc, and c-jun [18, 29]. One previous study showed that poorly differentiated NSCLC had low levels of AXIN1 relating a high expression of nuclear *β*-catenin, which was reversed in the well- or moderately differentiated tumors [30]. The oncogenic effects of *AXIN* monoallele mutations that lead to *β*-catenin stabilization result from the dominant negative activity [31]. Compared to the CC homozygote of the rs12921862 SNP, the CA/AA genotypes were associated with a significantly higher overall survival rate and implicated in a better prognosis of the BC patients, which might indicate a potential dominant negative protective effect of the latter in NMIBC patients. Furthermore, these results are consistent with the better prognosis of NMIBC patients compared to the MIBC.

Tumor stratification is used to grade cancers according to their malignancy: high-grade tumor classification normally represents a worse prognosis compared to that of the low grade. Studies have shown that the *AXIN1* rs1805105 polymorphism is correlated with the early tumor stages (I and II, modified by the Union for International Cancer Control) and the small tumor size (under 5 cm) of HCC [32] but increases the risk of developing advance-stage renal carcinogenesis (RCC III and IV) [33]. However, we found no significant association between the rs1805105 genotypes and tumor grade in our study, although the C allele carriers had a higher (albeit nonsignificant: 51.7% vs. 39.5%) frequency of patients with high-grade tumor compared to those with low-grade tumors. This indicated a nonsignificant tendency that the C allele may be correlated with the potential risk of developing higher-grade tumors among BC patients. Interestingly, the frequency of T allele carriers of the rs370681 SNP with high-grade tumors was significantly elevated than that with low-grade tumors, which indicated an association of this SNP with severe BC. These results are consistent with previous studies on HCC and RCC.

Bladder cancer is an older-susceptible cancer although the average age of the afflicted has decreased over these years. In this study also, we found that the CA/AA carriers of rs12921862 SNP had significantly higher frequencies of patients older than 64 years compared to those under 64 years, indicating that the A allele could be one of the risk factors of increased BC susceptibility in the older population.

A correlation between reduced *AXIN1* expression and tumor progression has been reported in esophageal squamous cell carcinoma, and several mutations and polymorphisms of *AXIN1* have been found in squamous cell tumors and cell lines [23, 34, 35]. Several recent studies have detected *AXIN1* gene sequence variations in subsets of ovarian endometrial adenocarcinomas and advanced prostate cancer, indicating new potentially relevant *AXIN1* mutations [23, 36]. Furthermore, Chimge et al. found that AXIN1 could be a potential target for the management of ER^+^ breast cancer [14]. Our study demonstrated the association of certain *AXIN1* gene polymorphisms with higher BC susceptibility and higher overall survival rate of NMIBC patients. Our results provide a new insight into potential predictive factors of BC progression and prognosis.

## 5. Conclusions

In conclusion, *AXIN1* SNPs are potential risk factors of BC susceptibility, rs370681 is associated with a severe form of BC, and rs12921862 is a significant forecast factor to BC prognosis. However, our study has several limitations. We did not analyze the expression levels of *AXIN1* in the patients' sera and tumor tissues. Also, we only studied a cohort of southwest China, whereas different ethnic populations differ in the types and frequencies of genetic polymorphisms. Therefore, further studies on larger and more diverse cohorts are needed to validate *AXIN1* SNPs as reliable markers to predict the progression and prognosis of bladder cancer.

## Figures and Tables

**Table 1:** Characteristics of the studied population.

Characteristics	Patients	Controls
Age at first diagnosis (mean ± SD)	63.76 ± 12.14	59.88 ± 11.32
Sex		
Male	250 (79.1%)	225 (53.7%)
Female	66 (20.9)	194 (46.3%)
Smoking status		
Smoker	163 (51.6%)	184 (43.9%)
Nonsmoker	153 (48.4%)	235 (56.1%)
Tumor stage		
MIBC	149 (47.2%)	
NMIBC	167 (52.8%)	
Tumor grade		
High grade	182 (57.6%)	
Low grade	134 (42.4%)	
Clinical stage		
I (Ta ~ T1N0M0)	156 (53.1%)	
II (T2N0M0)	84 (28.6%)	
III (T3N0M0, T4aN0M0)	34 (11.6%)	
IV (T4bN0M0, TnNnM0, TnNnMn, *n* ≥ 1)	20 (6.7%)	

**Table 2:** Primer sequences and reaction conditions for genotyping three tag SNPs in *AXIN1* gene.

SNPs	Primer sequence	Major/minor gene	Annealing temperature (°C)	Restriction enzyme	Product size (bp)
rs12921862	F: 5′-CTCACGCCAGTGCCTCTACT-3′	C/A	62	*ScrF I*	A: 216
	R: 5′-ATGCCATCCATGTGGAAACT-3′				C: 110 + 106

rs1805105	F: 5′-CTGGATACCTGCCGACCTTA-3′	T/C	65	*Fok I*	C: 245
	R: 5′-ACCTTTCCCTGGCTTGTTCT-3′				T: 186 + 59

rs370681	F: 5′-GAGGCCTAAGCTCCAGGCACT-3′	C/T	66	*Btsα I*	T: 166
	R: 5′-AAGGAAAGTGGGTTCTCCACCCA-3′				C: 150 + 16

**Table 3:** Distribution of SNPs in *AXIN1* between patients and controls as well as their association with bladder cancer risk.

Model	Genotype	Patients	Controls	Logistic regression	
		*N* (%)	*N* (%)	OR (95% CI)	*P* value
*rs12921862*					
Codominant	CC	205 (64.9%)	375 (89.5%)	1.00	
	CA	106 (33.5%)	37 (8.8%)	**5.24 (3.47-7.91)**	**<0.001**
	AA	5 (1.6%)	7 (1.7%)	1.31 (0.41-4.17)	
Dominant	CC	205 (64.9%)	375 (89.5%)	1.00	
	CA/AA	111 (35.1%)	44 (10.5%)	**4.61 (3.13-6.81)**	**<0.001**
Recessive	CC/CA	311 (98.4%)	412 (98.3%)	1.00	
	AA	5 (1.6%)	7 (1.7%)	0.95 (0.30-3.01)	0.93
Overdominant	CC/AA	210 (66.5%)	382 (91.2%)	1.00	
	CA	106 (33.5%)	37 (8.8%)	**5.21 (3.46-7.86)**	**<0.001**
Allele	C	516 (81.6%)	787 (93.9%)	1.00	
	A	116 (18.4%)	51 (6.1%)	**3.45 (2.44-5.00)**	**<0.001**
*rs1805105*					
Codominant	TT	168 (53.5%)	255 (60.9%)	1.00	
	TC	125 (39.8%)	136 (32.5%)	**1.40 (1.02-1.90)**	0.11
	CC	21 (6.7%)	28 (6.7%)	1.14 (0.63-2.07)	
Dominant	TT	168 (53.5%)	255 (60.9%)	1.00	
	TC/CC	146 (46.5%)	164 (39.1%)	**1.35 (1.00-1.82)**	**0.046**
Recessive	TT/TC	293 (93.3%)	391 (93.3%)	1.00	
	CC	21 (6.7%)	28 (6.7%)	1.00 (0.56-1.80)	1
Overdominant	TT/CC	189 (60.2%)	283 (67.5%)	1.00	
	TC	125 (39.8%)	136 (32.5%)	**1.38 (1.01-1.87)**	**0.04**
Allele	T	461 (73.4%)	646 (77.1%)	1.00	
	C	167 (26.6%)	192 (22.9%)	1.22 (0.96-1.55)	0.11
*rs370681*					
Codominant	CC	114 (37.9%)	204 (48.7%)	1.00	
	CT	152 (50.5%)	190 (45.4%)	**1.43 (1.05-1.96)**	**0.002**
	TT	35 (11.6%)	25 (6%)	**2.51 (1.43-4.40)**	
Dominant	CC	114 (37.9%)	204 (48.7%)	1.00	
	CT/TT	187 (62.1%)	215 (51.3%)	**1.56 (1.15-2.10)**	**0.004**
Recessive	CC/CT	266 (88.4%)	394 (94%)	1.00	
	TT	35 (11.6%)	25 (6%)	**2.07 (1.21-3.55)**	**0.01**
Overdominant	CC/TT	149 (49.5%)	229 (54.6%)	1.00	
	CT	152 (50.5%)	190 (45.4%)	1.23 (0.91-1.65)	0.17
Allele	C	380 (63.1%)	598 (71.4%)	1.00	
	T	222 (36.9%)	240 (28.6%)	**1.46 (1.16-1.82)**	**0.001**

Boldfaced values indicate a significant difference at the 5% level. OR: odds ratio; CI: confidence interval.

**Table 4:** Distribution of SNPs in *AXIN1* between patients' characteristics and their association with bladder cancer risk.

Characteristics	rs12921862			rs1805105			rs370681		
	Genotype		*P* value^a^	Genotype		*P* value^a^	Genotype		*P* value^a^
	CC	CA/AA		TT	TC/CC		CC	CT/TT	
Age									
≤64 years old	102 (70.8%)	42 (29.2%)	***0.04***	85 (59%)	59 (41%)	0.10	56 (40.3%)	83 (59.7%)	0.83
>64 years old	103 (59.9%)	69 (40.1%)		83 (48.8%)	87 (51.2%)		58 (35.8%)	104 (64.2%)	
Sex									
Male	164 (65.9%)	85 (34.1%)	0.77	128 (51.6%)	120 (48.4%)	0.47	88 (37.5%)	147 (62.5%)	0.96
Female	41 (61.2%)	26 (38.8%)		40 (60.6%)	26 (39.4%)		26 (39.4%)	40 (60.6%)	
Smoke									
Smoker	111 (68.1%)	52 (31.9%)	0.4	80 (49.4%)	82 (50.6%)	0.25	58 (37.7%)	96 (62.3%)	0.90
Nonsmoker	94 (61.4%)	59 (38.6%)		88 (57.9%)	64 (42.1%)		56 (38.1%)	91 (61.9%)	
Tumor stage									
MIBC	98 (65.3%)	52 (34.7%)	0.94	74 (49.7%)	75 (50.3%)	0.97	48 (33.1%)	97 (66.9%)	0.44
NMIBC	107 (64.5%)	59 (35.5%)		94 (57%)	71 (43%)		66 (42.3%)	90 (57.7%)	
Tumor grade									
High grade	120 (65.9%)	62 (34.1%)	0.49	87 (48.3%)	93 (51.7%)	0.18	56 (32%)	119 (68%)	***0.04***
Low grade	85 (63.4%)	49 (36.6%)		81 (60.5%)	53 (39.5%)		58 (46%)	68 (54%)	
Recurrence									
Recurrent	59 (63.4%)	34 (36.6%)	0.84	50 (53.8%)	43 (46.2%)	0.97	32 (36%)	57 (64%)	0.40
Nonrecurrent	146 (65.5%)	77 (34.5%)		118 (53.4%)	103 (46.6%)		82 (38.7%)	130 (61.3%)	
Metastasis									
Metastatic	31 (62%)	19 (38%)	0.68	24 (49%)	25 (51%)	0.70	19 (40.4%)	28 (59.6%)	0.07
Nonmetastatic	174 (65.4%)	92 (34.6%)		144 (54.3%)	121 (45.7%)		94 (37.1%)	159 (62.9%)	

Italic values indicate a significant difference at the 5% level. ^a^Adjusted by age, sex, smoking status, tumor stage, tumor grade, recurrence status, and metastasis status.

**Table 5:** Association between SNPs in *AXIN1* and overall survival for patients with MIBC or NMIBC.

SNP/genotype	MIBC			NMIBC		
	Alive/dead	HR (95% CI)^a^	*P* value^a^	Alive/dead	HR (95% CI)^a^	*P* value^a^
rs12921862						
CC	75/22			96/12		
CA	34/13			58/1		
AA	2/3			0/0		
Codominant		1.41 (0.83-2.42)	0.21		0.10 (0.01-0.84)	***0.03***
Dominant		1.45 (0.74-2.88)	0.28		0.10 (0.01-0.84)	***0.03***
Recessive		1.94 (0.53-6.89)	0.32		NA	NA
rs1805105						
TT	54/19			89/6		
TC	46/16			56/7		
CC	11/2			8/0		
Codominant		1.40 (0.71-2.76)	0.34		0.97 (0.34-2.72)	0.95
Dominant		1.21 (0.58-2.54)	0.62		1.12 (0.35-3.62)	0.85
Recessive		4.28 (0.88-20.88)	0.07		0.00 (0.00-NA)	0.99
rs370681						
CC	37/10			60/7		
CT	55/23			69/5		
TT	16/3			16/0		
Codominant		1.30 (0.72-2.34)	0.38		0.54 (0.19-1.54)	0.25
Dominant		1.76 (0.76-4.06)	0.18		0.60 (0.18-1.97)	0.40
Recessive		0.82 (0.21-3.17)	0.77		0.00 (0.00-NA)	0.99

Italic values indicate a significant difference at the 5% level. ^a^Adjusted by age, sex, smoking status, tumor stage, tumor grade, recurrence status, and metastasis status. HR: hazard ratio; CI: confidence interval.

## Data Availability

The data used to support the findings of this study are currently under embargo while the research findings are commercialized. Requests for data, 6 months after publication of this article, will be considered by the corresponding authors.
